# Dissecting the Repertoire of DNA-Binding Transcription Factors of the Archaeon *Pyrococcus furiosus* DSM 3638

**DOI:** 10.3390/life8040040

**Published:** 2018-09-21

**Authors:** Antonia Denis, Mario Alberto Martínez-Núñez, Silvia Tenorio-Salgado, Ernesto Perez-Rueda

**Affiliations:** 1 Facultad de Medicina, Universidad Juárez Autónoma de Tabasco, C.P. 86100 Tabasco, Mexico; denis.rose@gmail.com; 2 Facultad de Ciencias, Universidad Nacional Autónoma de México, Unidad Académica de Ciencias y Tecnología de la UNAM en Yucatán, Carretera Sierra Papacal-Chuburna Km. 5, C.P. 97302 Mérida, Yucatán, Mexico; 3 Tecnológico Nacional de México, Instituto Tecnológico de Mérida, C.P. 97000 Mérida, Yucatán, Mexico; s.tenorio.salgado@gmail.com; 4 Instituto de Investigaciones en Matemáticas Aplicadas y en Sistemas, Universidad Nacional Autónoma de México, Unidad Académica Yucatán, C.P. 97302 Mérida, Yucatán, Mexico

**Keywords:** transcription factors, DNA-binding domain, regulatory interaction, archaeon, regulatory network

## Abstract

In recent years, there has been a large increase in the amount of experimental evidence for diverse archaeal organisms, and these findings allow for a comprehensive analysis of archaeal genetic organization. However, studies about regulatory mechanisms in this cellular domain are still limited. In this context, we identified a repertoire of 86 DNA-binding transcription factors (TFs) in the archaeon *Pyrococcus furiosus* DSM 3638, that are clustered into 32 evolutionary families. In structural terms, 45% of these proteins are composed of one structural domain, 41% have two domains, and 14% have three structural domains. The most abundant DNA-binding domain corresponds to the winged helix-turn-helix domain; with few alternative DNA-binding domains. We also identified seven regulons, which represent 13.5% (279 genes) of the total genes in this archaeon. These analyses increase our knowledge about gene regulation in *P. furiosus* DSM 3638 and provide additional clues for comprehensive modeling of transcriptional regulatory networks in the *Archaea* cellular domain.

## 1. Introduction

*Archaea*, *Bacteria*, and *Eukarya* represent the three domains of life. Organisms included in the *Archaea* cellular domain are highly diverse in morphology, physiology, and natural habitats [1,2,3,4]. An interesting feature associated with Archaea is their basal transcription machinery, which resembles that of eukaryotes.

In this regard, Archaea include a purine-rich transcription factor B recognition element (BRE), which is recognized by the transcription factor TFIIB (TFB), immediately followed by a TATA box promoter sequence centered at a distance of 26/27 bp, upstream of the transcription start site (TSS) [5,6,7], a homologue of the transcription factor TFIIB (TFB), and an RNA polymerase that contains between 8 and 13 subunits [8,9]. To initiate transcription, TBP binds to TATA box, followed by the binding of TFB to the DNA-TBP complex by recognition of the BRE sequence [10] and, the recruitment of RNA polymerase [11]. In contrast, archaeal mRNAs and DNA-binding transcription factors (TFs) are structurally similar to their bacterial counterparts [12,13]. Therefore, this regulatory organization raises basic questions with regard to the mechanisms of transcriptional regulation and the manner by which bacteria-like TFs may interact or interfere with the components of the eukaryotic-like basal transcriptional machinery within an archaeal cell. It is for this reason that archaeal DNA-binding TFs represent an important class of proteins to explain the molecular mechanisms that underlie transcription regulation. Even though the ever-growing number of archaeal genome sequences reveals an increasing list of potential regulators [14,15], their transcriptional regulation begins to be documented, and the most detailed and advanced studies have been performed for a few TFs, mainly from the Lrp/AsnC family (formerly the Feast/Famine protein family) or recent studies described in *Halobacterium salinarum* and other methanogens [12,16,17,18,19].

In this regard, the archaeon *Pyrococcus furiosus* represents an interesting model to evaluate the archaeal regulatory elements. *P. furiosus* is an anaerobic and heterotrophic organism that grows with a temperature range between 70 °C and 103 °C. This archaeon has been used for the production of diols for various industrial processes, because of the resistance of their enzymes in laboratory processes [20]. Therefore, in the present study, an exhaustive bioinformatic analysis of the repertoire of TFs identified in *P. furiosus* was performed. This analysis allowed us to deduce the distribution of its TFs and their evolutionary families. Using this repertoire of TFs, we show that (1) 4% of the total genes encode TFs, reinforcing the notion that the genome of archaeal genomes encodes a low proportion of TFs as it was previously described [21] and being similar to bacteria described as intracellular pathogens, opportunistic pathogens and extremophiles (2) a considerable proportion of TFs entail one or two structural domains, contrasting to the observed in archaeal genomes, where most of TFs are monodomain, (3) seven regulons are present and were identified based on sequence inference and the published literature, being the first description in a global scale in this archaeaon, and (4) an overrepresentation of Sulfur and maltose metabolisms are associated with each regulon, among others.

## 2. Methods

### 2.1. Identification of DNA-Binding TFs

In order to identify and analyze the repertoire of TFs in the archaeon *Pyrococcus furiosus* DSM 3638, the complete genome was downloaded from the NCBI database. Then, we collected the probable regulatory proteins from P2TF [22] and the Transcription Factor DB [23], two databases that comprise computationally derived predictions of DNA-binding TFs by using the Superfamily library and PFAM hidden Markov models (HMMs). From this data set, around 20% of proteins annotated as transposases, invertases, and integrases were manually excluded. In brief, this exclusion was based on sequence comparisons against the National Center for Biotechnology Information’s nonredundant (NR) protein database (*E* ≥ 0.001) by conducting a BLAST search followed by the identification of protein domains via a Conserved Domain (CD) database search (*E* ≥ 0.001) [24].

Alternatively, 78 family-specific HMMs previously reported for the bacterium *Escherichia coli* K-12 [25] were used to scan the whole archaeon proteome sequence (*E* ≥ 0.001), with the *hmmsearch* module from the HMMer suite of programs (http://hmmer.org). In brief, these HMMs were constructed by [25] using the previously identified TF families of *E. coli* K-12 as seeds and considering every protein family’s DNA-binding domain (DBD) sequences (around 60 amino acids in length). Proteins with less than 50% coverage in the DNA-binding region relative to their corresponding HMM were excluded.

### 2.2. Structural Diversity Associated with TFs

Structural assignations to *P. furiosus* protein sequences were based on Superfamily annotations [26], whereas family assignations were based on information from the PFAM [27] and the CD databases [24,26,27,28], using the HMMer program. 

### 2.3. Functional Classes of the Regulated Genes

Regulated genes were classified according to their functional class based on arCOGs [29], KEGG [30], PFAM [27], and genome annotations.

### 2.4. Identification of DNA-Binding Sites

Pattern searches were achieved using the suite of programs in RSA tools (http://embnet.ccg.unam.mx/rsa-tools/). DNA-binding sites identified in the literature were used as seeds to scan the upstream region of each gene in *P. furiosus*, i.e., 400 bp upstream and 50 bp downstream in relation to the start site for translation initiation. All sites were retrieved, with 0 and 1 mismatch. Posteriorly, the DNA-binding sites were cross-checked against the sites previously identified, in order to evaluate their significance.

### 2.5. Enrichment Analysis of Functional Annotations

Enrichment analysis was computed using a hypergeometric test, as explained in reference [30]. The *p* values were corrected for multiple testing by using the method of Benjamini and Hochberg [30]. The functional classes were evaluated using KEGG annotations.

## 3. Results

### 3.1. Distribution and Domain Architecture of TFs

**Figure 1:**
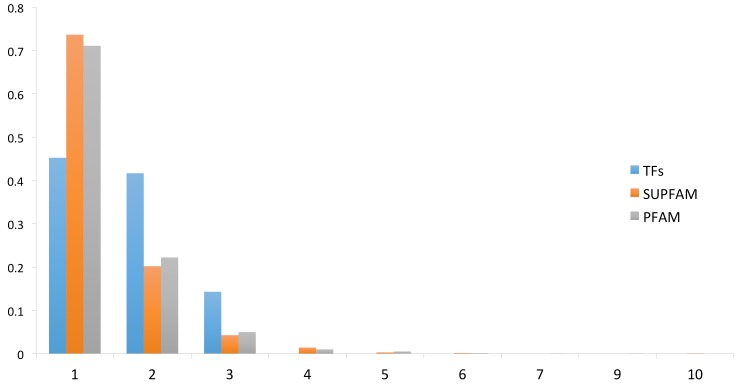
Domain organization of the DNA-binding transcription factors (TFs). On the *x*-axis is the proportion of proteins with different domains for the TFs and for the total proteins of *P. furiosus*. The numbers on the *y*-axis correspond to TFs with 1 domain, 2 domains, 3 domains, and so on.

To evaluate the distribution of DNA-binding TFs in *P. furiosus*, we used a two-step strategy. In the first step, we scanned two databases to identify potential DNA-binding TFs; for the second step, we used the sequences of recovered TFs to construct a battery of TF family-specific HMMs (see Methods for details) to scan the complete proteome of *P. furiosus*. These steps allowed for the detection of 86 proteins as probable TFs that represent the 4% of the total genes encoded in this archaeon. In a posterior step, the identified TFs repertoire was associated with its structural domains using the Superfamily database assignations in order to evaluate the domains architecture and its diversity. (Supplementary Table SI). (Figure 1). [31] From this assessment we identified that 38 (44%) TFs were monodomain, 35 (41%) have two domains, and 13 (15%) TFs contain three structural domains. These proportions (44%, 41%, and 15%) were statistically significant in the set of TFs (Wilcoxon test, *p* = 0.022490). In order to evaluate if there is an overrepresentation of proteins with multiple structural domains, the complete proteome of *P. furiosus* was evaluated in terms of its structural domain organization, considering the Superfamily assignments and then compared to domain architecture of TFs. From this, we determined that 73% of the total proteins in *P. furiosus* have one domain, 20% have two domains, 4% have three domains, and 3% have four or more structural domains. From this comparison, we found that a small proportion of proteins with one structural domain were associated with TFs among the complete proteins of *P. furiosus.* In contrast, there was a greater proportion of TFs with two or three domains relative to the total proteins of this archaeon, suggesting a bias towards multidomain proteins in the set of TFs. The multidomain architecture present in the TFs of *P. furiosus*, and probably in all Archaea organisms, may be due to a mechanism that increases the sensing of diverse signals and activate the regulatory response in *P. furiosus*. This found is similar to the observed in all the bacteria genomes. In particular, in TFs of *E. coli* K-12 there is a predominance of two-domain proteins (~75%) followed by three-domain proteins (~12%), single-domain proteins (~10%) and, finally, four-domain proteins (~3%) [25,32]. Finally, although we considered that the number of TFs identified in this archaeon could be close to the total number of DNA-binding regulatory proteins that *P. furiosus* needs to regulate its complete repertoire of genes, the existence of new DNA-binding structures cannot be discarded. In addition, the existence of alternative regulatory mechanisms, such as riboswitches [33] and DNA curvature [34], could also influence gene expression where there is no evidence of regulation mediated by TFs.

### 3.2. Protein Domain and Regulatory Families Associated with the Repertoire of TFs

**Figure 2:**
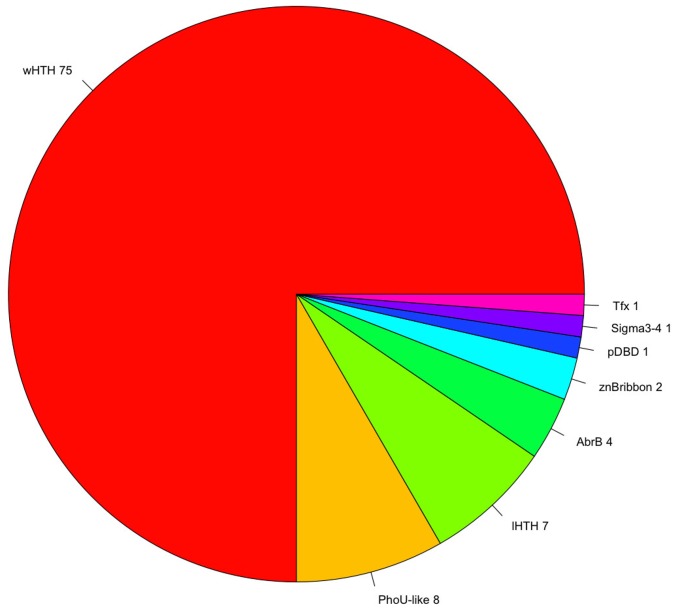
Repertoire of DNA-binding TFs. Diversity of DNA-Binding Domains (DBDs) associated with *P. furiosus*. The winged helix-turn-helix (wHTH) domain represents 73% of the total repertoire of DBDs; the PhoU-like and lambda HTH domains each represent 6% of the total. In minor proportions are alternative DBDs, such as the AbrB, AlbA, and zinc beta ribbon domains, among others, and these represent 12% of the total of DBDs.

A fundamental aspect associated with TFs is protein domain organization. The structural organization provides important clues concerning how they coordinate gene regulation, depending on their ability to recognize molecular cognates, such as small molecules, DNA, or other proteins. In this regard, the repertoire of TFs previously identified was analyzed using the library of HMMs deposited in the Superfamily database [35]. From this analysis, we identified that the most abundant DNA-Binding Domain (DBD) is the winged helix-turn-helix (wHTH) domain, which was detected in 73% of the total TFs (Table 1). The second and third most abundant binding domains corresponded to the PhoU-like and lambda repressor-like domains, each one detected in 7% of the total TFs. This found correlates with previous analysis [32,36,37] that described that up to 84% of the DNA-binding domains (DBDs) in one-component TFs comprise a DNA-binding helix–turn–helix (HTH), and in particular the winged helix DNA-binding domain (wHTH), representing around 45% of the total set of TFs contains this domain. In addition, these proportions are also similar to the regulatory proteins identified in the bacterium *Escherichia coli* K-12, where the wHTH corresponds to the 36% of the total structures associated to the [25]. Therefore, this result, reinforces the notion of common ancestry in the transcriptional regulatory machinery of *Bacteria* and *Archaea* [38,39]. Alternative DBDs, such as the integration AbrB, and putative DBDs were also identified, although both types were found in lower proportions (corresponding to around 12% of the total TFs) (Figure 2). This information suggests constraints in the diversity associated with DBDs, where the most predominant structure is the wHTH domain, and a subsequent diminished diversity of alternative DBDs.

**Figure 3:**
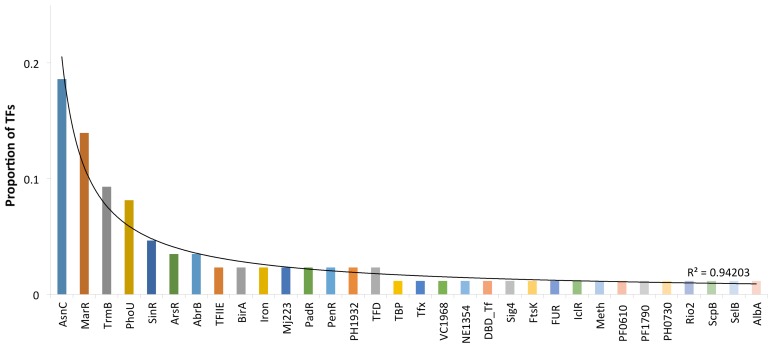
TF families identified in *P. furiosus*. The *x*-axis indicates the family names; on the *y*-axis are proportions of the regulatory families.

Another question associated with DBDs concerns the regulatory families associated with *P. furiosus*. In this regard, families are associated at the upper hierarchical level, the superfamilies or DBDs described above. Thus, most TF families have been found to undergo lineage-specific duplications that result in the accumulation of particular families in some microbial species, such as the LysR family in *E. coli* or AraC/XylS in *Staphylococcus aureus* [25,40]. In *P. furiosus*, we identified 31 different evolutionary families based on their DBDs (Supplementary Table SI). Among these, the AsnC (19%) and MarR (14%) families represented 33% of the total regulatory proteins identified in this archaeon (Figure 3). Both families are associated with the wHTH superfamily. The next two families, TrmB (9%), and PhoU (8%), represent 17% of the collection and are associated with the wHTH and PhoU superfamilies, whereas the other 28 families comprised 50% of the collection, containing a small number of members per family. In this regard, previous analyses [41,42] suggested that global regulators (GRs) in *Archaea* could be associated with large families [43], contrary to the GRs identified in small families in *Bacteria*. In this regard, the AsnC, MarR, TrmB, and PhoU familes were the most populated, being candidates to include GRs. Therefore, all these data suggest that a large number of duplication events associated with successful families are the main forces governing the increase in the regulatory repertoire, and this is consistent with the more general notion that a genome evolves from a set of precursor genes to a mature size via gene duplications and increasing modifications [44]. Therefore, the domain organization and more generally the properties of the TF repertoire described here allow for evaluation of whether the organization and evolution of regulatory networks in *P. furiosus*, in particular in this archaeon, are similar to those observed in other biological systems, such as bacteria or yeast [45,46].

### 3.3. Regulons Identified

In order to identify those genes regulated by the complete repertoire of TFs in *P. furiosus*, we conducted an exhaustive literature search for articles associated with the identified regulators. Based on these searches, we identified evidence of regulated genes for seven different TFs. These regulated genes were used as seeds to expand the probable regulon by considering two criteria: an expansion taking into account those genes with similar DNA-binding sites and previously identified as associated with each transcriptional regulator; and those genes clustered in transcription units (operons) retrieved from Prokaryotic Operon Database [47], i.e., if the regulated gene belonged to one operon and the related genes were not included in the original set, they were posteriorly added. From this search, 279 genes were considered probable members of seven regulons: 76 genes were identified from literature and databases searches, 13 from pattern searches, and 185 were associated via operon predictions. (Supplementary Figure S1 and Table SII). This set of regulatory and regulated genes in *P. furiosus* opens the opportunity to understand the gene regulation in this archaeon and probably in other organisms of the same taxonomical division. In what follows, we describe the regulons identified and their corresponding functions.

### 3.4. Iron-Dependent Repressor Regulon (PF0851)

**Figure 4:**
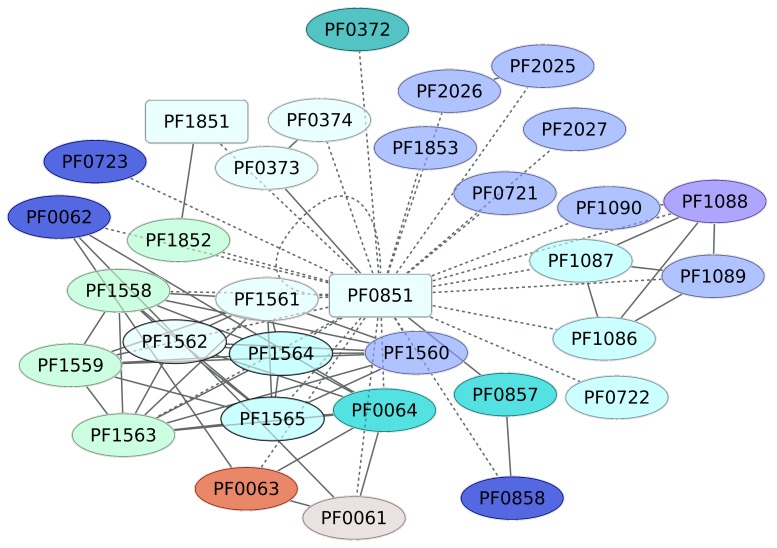
Genes associated with the Iron-dependent repressor family (PF0851). In red squares are the regulatory proteins identified in this work. The continuous line indicates those genes for which there is experimental evidence of regulation, whereas dashed lines correspond to predicted interactions based on analysis using the String server (interaction values 0.7 were considered) (http://string.embl.de/). Colors for genes correspond to COG categories. TFs are in rectangles; inferred TFs (not based on experimental evidence) are in green. Other categories and colors are as follows: K category, blue; G, yellow; C, dark green; P, dark blue; E, purple; O, red. Shown in white are those genes with no COG assignment or with a hypothetical or general function.

The transcriptional regulator PF0851, an iron-dependent repressor regulon, has been classified as a member of the DtxR family, where a second TF is included (PF1179, an uncharacterized protein). PF0851 probably regulates the expression of 32 different genes, which are associated with riboflavin metabolism (PF0064, PF0063 or RibH, PF0062, and PF0061) according to KEGG pathway 00740 (Riboflavin metabolism) (Figure 4). Indeed, this pathway is enriched in the data set, with a *p* value of 5.39 × 10^−5^. Another group of genes regulated by the iron-dependent repressor regulon corresponds to those associated with RNA polymerase (KEGG pathway 03020), which was enriched in the data set, with a *p*-value of 0.000834. Finally, PF0851 regulates its own expression and is a putative transcriptional regulator (PF1851) and a member of the SinR family. These findings suggest that the regulon probably involves a regulatory cascade associated with iron transport and assimilation, among other processes.

### 3.5. Maltose and Trehalose Metabolism (PF1743)

**Figure 5:**
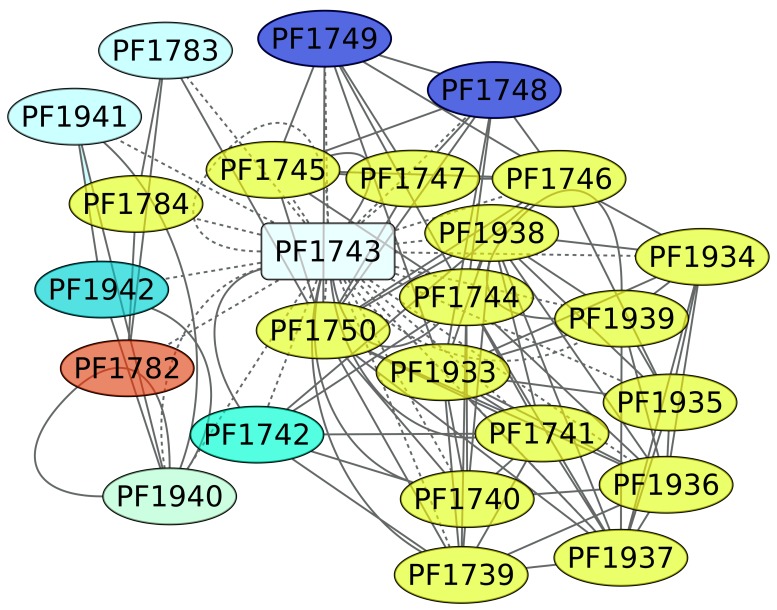
Genes associated with the maltose and trehalose metabolism regulon (PF1743). The color codes are the same as Figure 4.

TrmB (PF1743) is a TF involved in the expression of genes devoted to maltose and trehalose metabolism. In this regard, it is interesting that almost 65% of the genes under the regulation of TrmB are involved in carbohydrate transport and metabolism (Figure 5). The ABC transporters of KEGG pathway 02010 are enriched, with a *p*-value of 2.07 × 10^−6^. In addition, when we evaluated the structural domain assignment, the PFAM associated with the binding protein-dependent transport system inner membrane component (PF00528) is overrepresented in the data set (*p*-value of 0.00021), reinforcing the notion that the genes under the control of TrmB are mainly associated with carbon source uptake.

### 3.6. TrmBL1 Regulon (PF0124)

**Figure 6:**
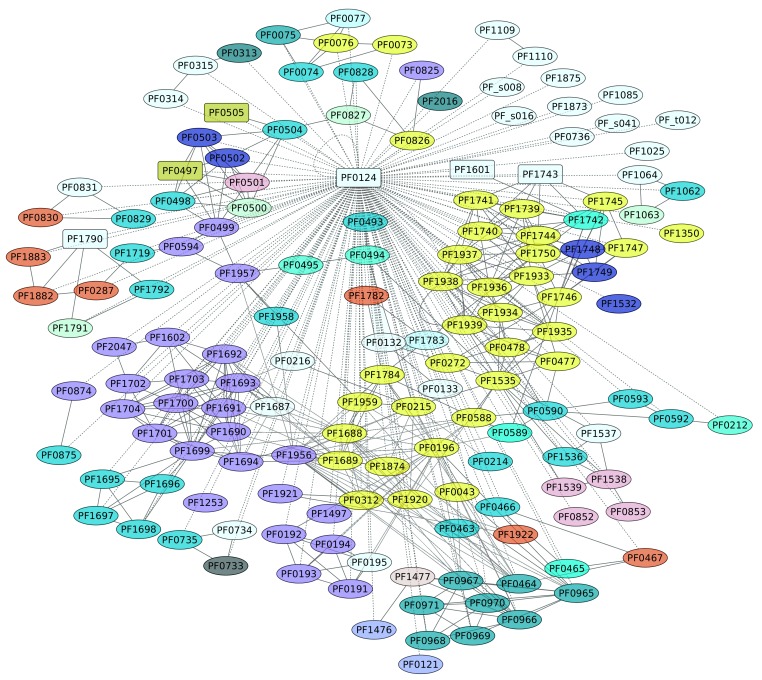
Genes regulated by TrmBL1 (PF0124). The color codes are the same as Figure 4.

The genome of *P. furiosus* contains eight members of the TrmB family. TrmBL1 (PF0124) has been characterized as a transcriptional regulator of the genes encoding enzymes of the glycolytic pathway and the maltodextrin (MD) ABC transporter [48], and it also activates genes encoding gluconeogenic enzymes. At ambient temperatures, TrmBL1 behaves as a tetramer, whereas in the presence maltotriose or maltose TrmBL1 forms octamers [48]. In all cases, this TF recognizes a DNA-binding site downstream of the BRE-TATA box and overlaps the transcription start site on each promoter [49], a conserved sequence (*Thermococcales* glycolytic motif) [48,50,51]. Based on the information collected in this work, TrmBL1 can be considered a global regulator, because this TF influences the expression of 159 different genes (representing 57% of the complete collection of regulated genes) in a direct or indirect fashion (Figure 6). In this regard, five TFs are also under the control of TrmBL1: TrmBL1; two uncharacterized proteins, PF0497 and PF0505; another member of the TrmB family, PF1743; and PF1790, a transcriptional regulatory protein that belongs to the ArsR family. Indeed, a functional enrichment analysis showed that the KEGG pathways Carbon metabolism (ID 01200) and Microbial metabolism in diverse environments (ID 01120) are enriched, with *p*-values of 1.08 × 10^−5^ and 1.72 × 10^−5^, respectively. Figure 6 shows the diversity of functions associated with this regulon, such as carbohydrate and amino acid transport and metabolism, energy production and conversion, and others. In this regard, the diversity of functions associated with this regulon, together with the collection of TFs under the control of this transcriptional regulator, suggest that TrmBL1 is a global regulator in *P. furiosus*.

### 3.7. Phr Regulon (PF1790)

**Figure 7:**
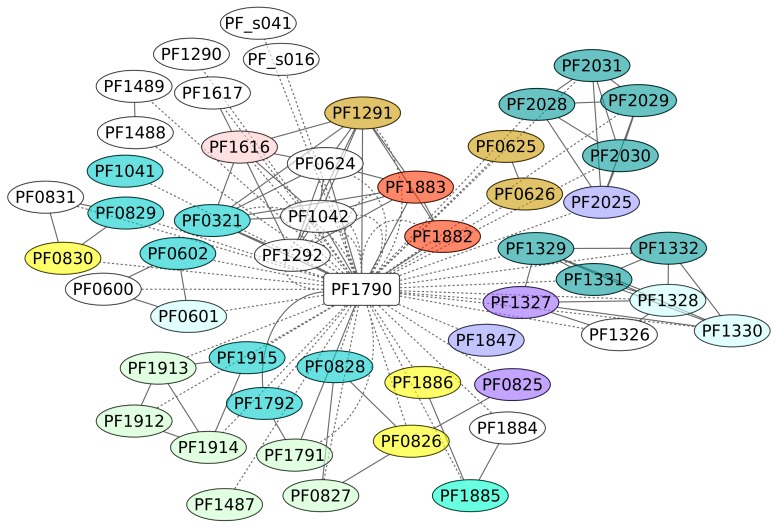
Phr regulon (PF1790). The color codes are the same as Figure 4.

The TF Phr (PF1790) is a member of the ArsR family of repressor proteins and has been described as a regulator of the heat shock response [52]. The overall structure of this protein has been identified as conserved among euryarchaeotal organisms [52] and shows a molecular chimera with significant folding similarity of its DBD to the bacterial SmtB/ArsR family, while its C-terminal portion was found to be a remote homologue of the eukaryotic BAG domain [53]. In this work, we identified 52 genes under the control of this protein (Figure 7). These regulated genes were classified under diverse functional groups, such as energy production and conversion and translation; however, an enrichment analysis showed that genes associated with the KEGG pathway for Sulfur metabolism (ID 00920) are enriched in the data set, with a *p*-value of 0.0133. We suggest that, similar to other regulons associated with stress conditions, such as RpoS of the bacterium *E. coli* K-12, their functions are heterogeneous, mainly because the cell requires functional diversity to contend against varied environmental conditions [54]. An interesting case associated with this regulon is the sulfhydrogenase 2 subunit beta (PF1329) gene, a system involved in the sulfur reductase enzyme. In addition, Phr bound to a 29-bp DNA sequence that overlaps the transcription start site. Three sequences conserved in the binding sites of Phr, TTTA at −10, TGGTAA at the transcription start site, and AAAA at position +10, were required for Phr binding and are proposed as consensus regulatory sequences of *Pyrococcus* heat shock promoters. It seems that almost all the genes associated with the Phr region are repressed, according to the experimental evidence and the position of the DNA-binding site [52,55].

### 3.8. Sur Regulon (PF0095)

**Figure 8:**
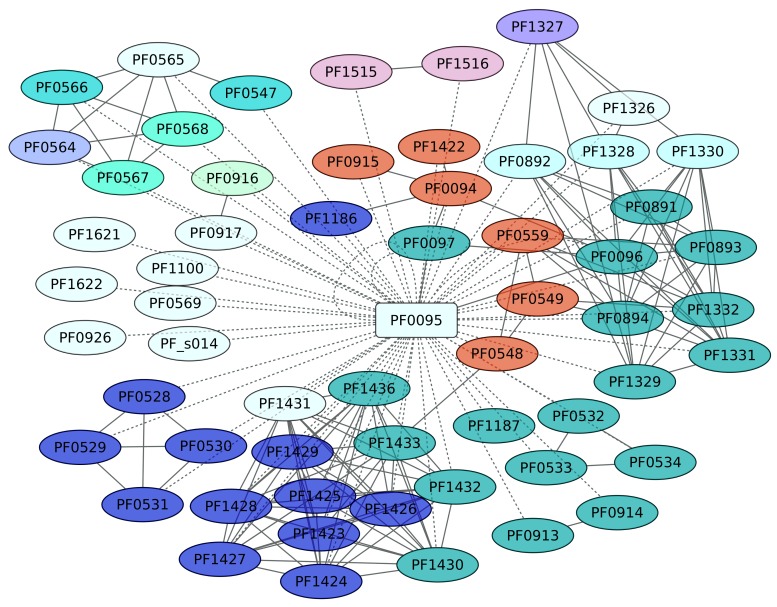
Sur regulon (PF0095). The color codes are the same as Figure 4.

SurR (PF0095) regulates hydrogen production in *P. furiosus* by a sulfur-dependent redox regulating switch [56]. *P. furiosus* has been described as an organism that produces H_2_ during fermentation but undergoes a metabolic shift to produce H_2_S when elemental sulfur [S(0)] becomes available. SurR plays a central role in the primary response to S(0), activating the hydrogenase operons and repressing another set of genes, including the gene encoding sulfur reductase. In this work, 59 genes were identified as members of the SurR regulon; from these, we only found evidence of positive regulation for 7 genes, whereas 13 genes are negatively regulated (Figure 8). SurR recognizes a specific DNA-binding motif, GTTn(3)AAC [57]. One interesting result is the regulation of genes devoted to cobalt and cadmium extrusion, and also to sulfur metabolism. Indeed, the genes identified as playing a central role in the Sulfur metabolism pathway (KEGG ID 00920) are enriched, with a *p* value of 1.9 × 10^−8^, in the data set.

### 3.9. LrpA Regulon (PF1601)

LrpA (PF1601) [58,59] is a member of the AsnC family, the largest family of transcriptional regulators in *P. furiosus*, for which 16 members have been identified. This regulator has been described as a repressor of its own transcription, binding to a 46-bp sequence that overlaps the transcriptional start site of its own promoter [58]. We did not find enough evidence to associate this transcriptional regulator with additional metabolic processes.

### 3.10. TBP (PF1295)

The TBP (PF1295) has been described as a general factor that plays a role in the activation of archaeal genes transcribed by RNA polymerase. This protein binds to the TATA box promoter element, which lies close to the position of transcription initiation [60], and regulates the glutamate dehydrogenase gene (PF1602). We did not find evidence in the literature concerning its probable regulated genes.

## 4. Discussion

In this work, we compiled and analyzed a repertoire of DNA-binding TFs and regulated genes in *P. furious* DSM 3638, leading us to clues about the regulatory network organization of this archaeon. In general, we determined that around 4.5% of the genes in *P. furious* are devoted to regulation of gene expression, and almost 15% of genes in this archaeon are under the regulation of seven different TFs. Based on these data, TrmB1 must be considered a global regulator, because the large proportion of genes regulated (159 genes, or 56%) where seven probable TFs are included. Therefore, this global regulator could be influencing the gene expression of a large number of elements, mainly those involved in carbon assimilation (See Figure S2). In addition, we found a large proportion of regulatory proteins with structures similar to bacterial TFs, with a predominance of the wHTH motif. We also found that AsnC is the most abundant family identified so far in *P. furiosus*; MarR is the second most abundant, and TrmB is the third most abundant. Another observation extracted from our analysis is the multidomain composition of TFs, which contrasts with the overrepresentation of monodomain proteins associated to the total repertoire of proteins of *P. furiosus*. This multi-domain architecture on regulatory proteins could serve to the organism for the sensing of very diverse signals, which allow it to respond to changes originated in its extreme habitat of life. Altogether, this analysis provides new clues about the *P. furiosus* genetic regulation network; additionally, our findings can be expanded to other organisms.

## Figures and Tables

**Table 1:** Superfamilies and families associated with the total repertoire of TFs in *P. furiosus*.

Superfamily	Family(s)	Total of Proteins
wHTH	AsnC, MarR TrmB, ArsR, TFIIE, PenR, PadR, Iron, PH1932, Mj223, BirA, TFIIE, PenR, PadR, Iron, PH1932, Mj223, BirA, IclR, ScpB, Rio2, PH0730, PF1790, PF0610, Meth, FtsK, Fur, SelB	63
PhoU-like	PhoU	7
lHTH	SinR, VC1968, NE1354	6
AbrB	AbrB	3
znBribbon	TFD	2
pDBD	DBD_Tf	2
Tfx	Tfx	1
Sigma3,4	Sig4	1
AlbA	AlbA	1
TBP	TBP	1

## Supplementary Materials

The following are available online at http://www.mdpi.com/2075-1729/8/4/40/s1, Figure S1: Regulatory network associated with *P. furiosus*, Figure S2: Functional assignments based on COG annotations. Heat map representing the proportion of genes per functional category, Table SI: Assignation of superfamily domains for the collection of TFs, Table SII: Complete collection of TFs and target genes compiled and identified in Pyrococcus furiosus DSM 3638, Table SIII. Operons predicted in the genome of Pyrococcus furiosus DSM 3638.
