# Supplementary figures and images for: Dissecting the Repertoire of DNA-Binding Transcription Factors of the Archaeon Pyrococcus furiosus DSM 3638
Source: Life (Basel). 2018 Sep 21;8(4):40. doi: 10.3390/life8040040 (PMC6316755; doi:10.3390/life8040040)

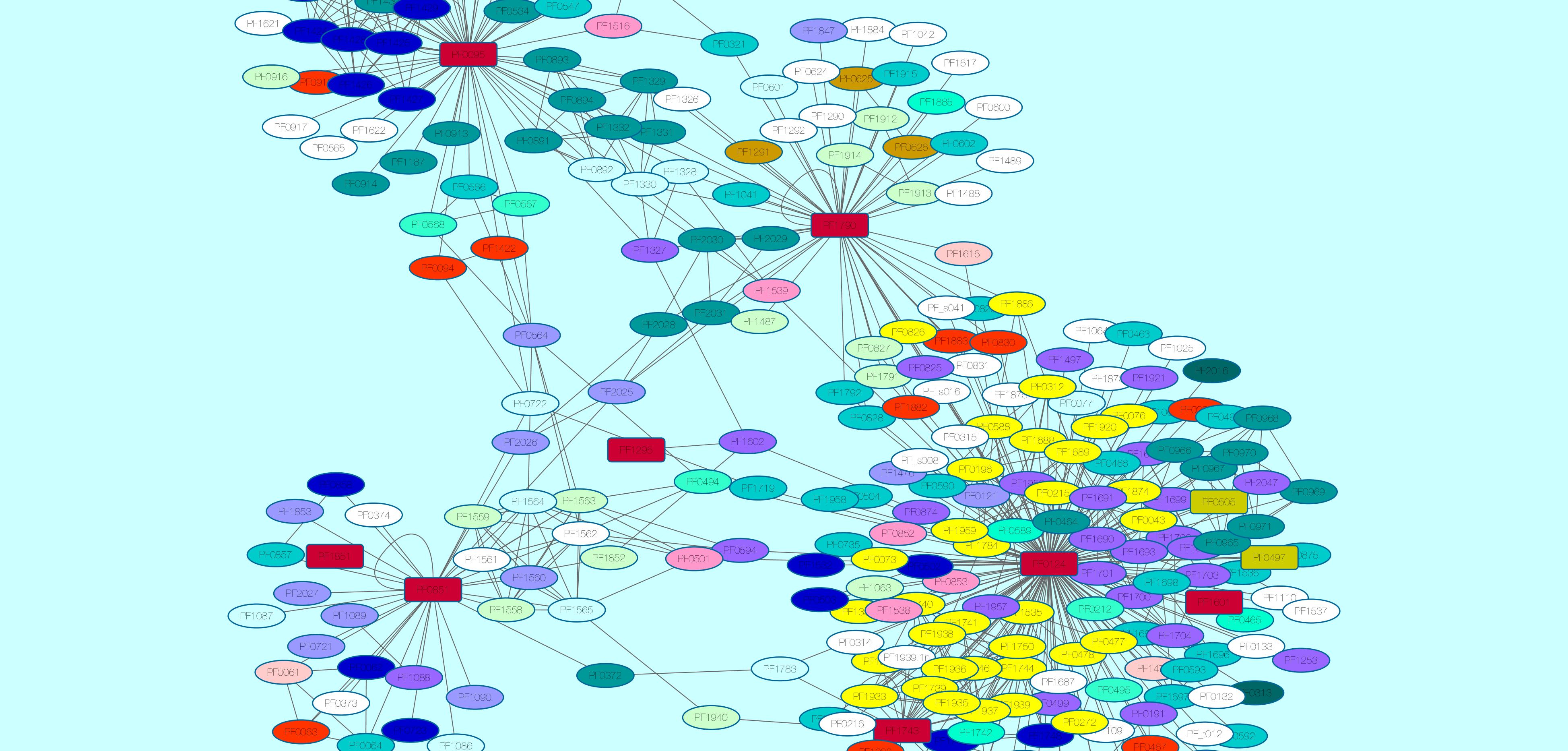

Supplement: Supplementary file 1 [file life-08-00040-s001.zip › supplement material/FigureS1_regulatory_network.jpg]

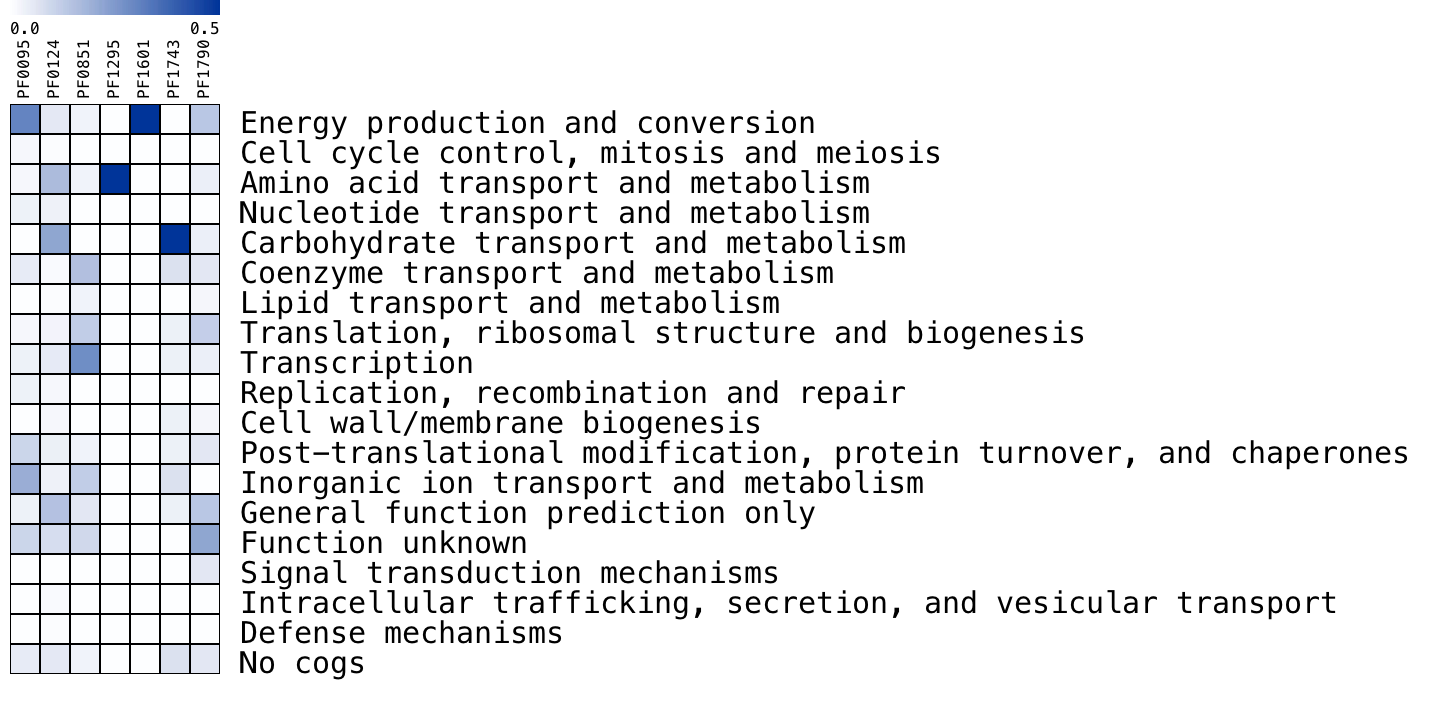

Supplement: Supplementary file 1 [file life-08-00040-s001.zip › supplement material/FigureS2_Pfuriosus_COGs.tif]
